# Bending and Buckling of Circular Sandwich Plates with a Hardened Core

**DOI:** 10.3390/ma14164741

**Published:** 2021-08-22

**Authors:** Zizi Pi, Zilong Zhou, Zongbai Deng, Shaofeng Wang

**Affiliations:** 1 School of Resources and Safety Engineering, Central South University, Changsha 410083, China; zizipi@csu.edu.cn (Z.P.); zlzhou@csu.edu.cn (Z.Z.); 2 College of Aerospace Engineering, Nanjing University of Aeronautics and Astronautics, Nanjing 210016, China; 185501012@csu.edu.cn

**Keywords:** circular sandwich plates, annular sandwich plates, hard core, Reissner’s theory, bending, stability, finite element analysis

## Abstract

Hard-core sandwich plates are widely used in the field of aviation, aerospace, transportation, and construction thanks to their superior mechanical properties such as sound absorption, heat insulation, shock absorption, and so on. As an important form, the circular sandwich is very common in the field of engineering. Thus, theoretical analysis and numerical simulation of bending and buckling for isotropic circular sandwich plates with a hard core (SP-HC) are conducted in this study. Firstly, the revised Reissner’s theory was used to derive the bending equations of isotropic circular SP-HC for the first time. Then, the analytic solutions to bending deformation for circular and annular sandwich SP-HCs under some loads and boundary conditions were obtained through the decoupled simplification. Secondly, an analytic solution to bending deformation for a simply supported annular SP-HC under uniformly distributed bending moment and shear force along the inner edge was given. Finally, the differential equations of buckling for circular SP-HCs in polar coordinates were derived to obtain the critical loads of overall instability of SP-HC under simply supported and fixed-end supported boundary conditions. Meanwhile, the numerical simulations using Nastran software were conducted to compare with the theoretical analyses using Reissner’s theory and the derived models in this study. The theoretical and numerical results showed that the present formula proposed in this study can be suitable to both SP-HC and SP-SC. The efforts can provide valuable information for safe and stable application of multi-functional composite material of SP-HC.

## 1. Introduction

The sandwich structure used in engineering generally refers to a composite structure consisting of two high-strength thin plates and sandwich materials filled between them [1,2,3,4]. The sandwich structure, featuring light mass, high stiffness, and high strength, has the properties of heat insulation, heat preservation, sound insulation and noise reduction, shock absorption, and fire prevention [5,6]. In addition, the sandwich structure avoids large area riveting in the production process, which weakens the stress concentration and thus greatly enhances the fatigue resistance strength of the structure. Consequently, the sandwich structure has been considered as an important structural material in aerospace engineering, and has been widely used in shipbuilding, train manufacturing, building structures, and other fields in recent years [7].

Many scholars have conducted in-depth studies on the mechanical behavior and properties of sandwich structures, and proposed many analysis and calculation models. In terms of linear theory alone, three main theories have been formed [8]. (a) Reissner’s theory [9], which only considers the antisymmetric deformation of sandwich plates and is proposed on the basis of the shear correction theory of Reissner sandwich plates. The theory treats the sandwich plate as a thin film and ignores its bending stiffness. Moreover, it considers that the stress component parallel to the plate plane in the sandwich is zero because the sandwich is soft. This theory is relatively simple in solving the overall bending and stability problems of sandwich plates, and its calculation can basically meet the requirements of engineering applications. (b) Hoff theory [10], which differs from Reissner’s theory only in that it is based on the assumption that the sandwich plate is a thin plate, i.e., the bending resistance and the transverse shear deformation of the plate are considered. Compared with that of Reissner’s theory, the equation derived from such a theory has an additional coefficient of bending stiffness Df of the surface board, as well as a different expression for the shear stiffness C. (c) Prusakov–Du Qinghua theory [11], a theoretical analysis model considering transverse elastic deformation of the sandwich proposed by Prusakov and Du Qinghua, who considered that, in practice, sandwich plates have not only antisymmetric bending deformation and overall instability deformation, but also symmetric deformation and local forms of instability. Unlike the Hoff theory, this theory takes into account the symmetric deformation of the sandwich plate. The materials considered in the above three theories have the following in common: sandwich plates are isotropic materials, with small deflection bending deformation, and the core of the sandwich plate is soft, that is, the sandwich only withstands the transverse shear force. Subsequently, a number of scholars have also studied orthotropic and anisotropic sandwich plates. Wang Zhenming and Dai Fuling explored the problems in deformation, stability, and vibration of orthotropic four-sided simply supported multilayered, sandwiched, and reinforced rectangular flat shells in terms of the shear deformation in the thickness direction [12]. OT Thomsen put forward an approximate analysis of the local buckling effect of orthotropic sandwich plates under local loads, did analytical calculations for specific cases, and compared them with the results of finite element analysis, which showed that the modularity ratio and the thickness of the bearing surface have a great influence on the local buckling effect [13]. T.S. Lok and Q.H. Cheng studied the bending and dynamic response of sandwich plates by equating them to orthotropic thick plates using dynamic equivalence and other methods [14,15]. Hoff theory and Prusakov–Du Qinghua theory are all classified as the first-order shear deformation theory. This theory considers that the deformation of each layer of the sandwich plate varies linearly along the thickness direction, which is not applicable to the sandwich plate problems with large differences in the stiffness of each layer, and it is also not applicable to the problems requiring high accuracy of shear stress between layers. In order to solve problems of such a sandwich plate, it is necessary to use the higher-order shear theory. B.N. Pandya and T. Kant assumed that the displacement pattern varies nonlinearly within the plane along the thickness direction of the plate and the transverse displacement is constant, thus giving a simple equivalent finite element method for a symmetrical multilayer plate [16]. Sokolinsky et al. investigated the buckling behavior of sandwich plates under different boundary conditions and boundary loads based on the higher-order shear theory [17].

Scholars at home and abroad have conducted many studies on the stability of sandwich structures, as instability of sandwich structures is an important form of structural failure. Unlike single-layer structures, sandwich structures produce both local buckling and overall buckling forms under in-plane loading. In the study of local buckling of sandwich structure plates, Heath [18] assumed the core layer as the elastic support of the sandwich structure plates and approximated the critical load for buckling of the sandwich structure plates. Aiello [19] et al. also studied the buckling of sandwich plates using similar assumptions to Heath and using the first-order shear theory. In terms of overall buckling research, Hadi [20] et al. investigated the overall buckling of sandwich plates based on the zig-zag model, and further analyzed the role of relevant parameters on the overall buckling. All the above researchers only consider the local buckling or overall buckling of sandwich plates alone, and do not consider the interaction between them. Such a research idea is applicable to SP-HC. However, for the sandwich plate with a soft core (SP-SC) to have significant transverse compressibility, the overall and local buckling have a certain degree of interaction and may even undergo mutual transformation. Therefore, Frostig [21] and Sokolinsky [17] analyzed the stability problem of the SP-SC by means of higher-order shear theory and analyzed their buckling behavior as influenced by boundary conditions. Dawe [22] and Yuan [23] investigated the local buckling and overall buckling of the sandwich plate by finite element methods with B-splines. Pandit et al. [24] improved the zig-zag higher-order theory and used this model to investigate the buckling properties of sandwich plates.

From the above research analyses, it can be found that the previous studies on the bending and stability of the sandwich structure mainly focus on the soft-core sandwich structure, which ignores the in-plane stiffness of the core. With the emergence and development of new materials, some hard-core sandwich structures having in-plane bending stiffness have emerged in engineering applications. Although some scholars have started to study the bending and stability of hard-core sandwich structures, there is a lack of theoretical analysis models for solving the bending and stability problems of the circular SP-HC in engineering. Therefore, we derive the equilibrium equations for bending of isotropic circular SP-HCs in a polar coordinate system, and give the corresponding expressions for internal forces and boundary conditions in this paper. For the common load forms and boundary conditions in engineering, the analytical forms of bending of circular and annular SP-HCs under various load forms and boundary conditions are given in this paper. In addition, we derive the critical loads for instability under in-plane loading of the SP-HC simply supported and fixed-end supported at its periphery. The analytical models proposed in this study can provide some valuable information and theoretical supports for analyzing the bending and stability of SP-HC structures.

## 2. Bending of the Circular SP-HC

**Figure 1:**
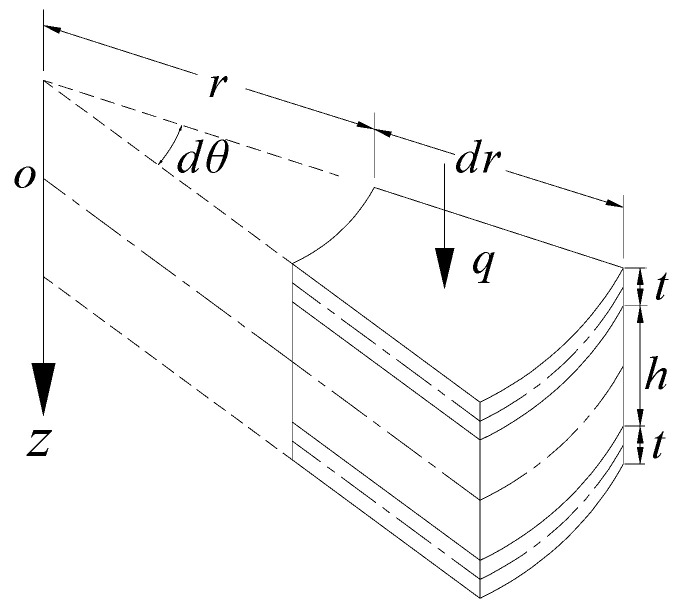
Coordinates and dimensions of the circular sandwich plate.

There is a circular SP-HC with plate thickness *t* and core thickness *h*. The materials of both plates and cores are isotropic, with modulus of elasticity *E_f_* and *E_c_*, and Poisson’s ratio *μ_f_* and *μ_c_*, respectively, as shown in Figure 1. A more convenient way to solve the circular plate problem is to use the polar coordinate system. For this purpose, the basic equation of the SP-HC represented by the rectangular coordinate system, which was derived by Ma and Deng based on the revisions for the soft sandwich hypothesis of Reissner’s theory from consideration of influence of transverse shear by first-order shear deformation theory [25,26,27], should be transformed into the polar coordinate. In polar coordinates, the internal force for bending of the circular SP-HC is shown in Equation (1).

(1) $$\begin{array}{l} M_{r}=-\left(D+D_{c}\right)\frac{\partial \psi_{r}}{\partial r}-\left(D\mu_{f}+D_{c}\mu_{c}\right)\left(\frac{1}{r}\psi_{r}+\frac{1}{r}\frac{\partial \psi_{\theta }}{\partial \theta }\right)\\ M_{\theta }=-\left(D+D_{c}\right)\left(\frac{1}{r}\psi_{r}+\frac{1}{r}\frac{\partial \psi_{\theta }}{\partial \theta }\right)-\left(D\mu_{f}+D_{c}\mu_{c}\right)\frac{\partial \psi_{r}}{\partial r}\\ M_{r\theta }=-\left(D\frac{1-\mu_{f}}{2}+D_{c}\frac{1-\mu_{c}}{2}\right)\left(\frac{1}{r}\frac{\partial \psi_{r}}{\partial \theta }-\frac{1}{r}\psi_{\theta }+\frac{\partial \psi_{\theta }}{\partial r}\right)\\ Q_{r}=C\left(\frac{\partial w}{\partial r}-\psi_{r}\right)\\ Q_{\theta }=C\left(\frac{1}{r}\frac{\partial w}{\partial \theta }-\psi_{\theta }\right) \end{array}$$

where

(2) $$D=\frac{E_{f}{\left(h+t\right)}^{2}t}{2\left(1-\mu_{f}^{2}\right)}$$

(3) $$D_{c}=\frac{E_{c}h^{3}}{12\left(1-\mu_{c}^{2}\right)}$$

(4) $$C=\frac{E_{c}h}{2\left(1+\mu_{c}\right)}=G_{c}h$$

where *D* is the combined bending stiffness of the circular sandwich plate; *D_c_* and *C* are the bending stiffness and shear stiffness of the core layer, respectively; *μ_f_* and *μ_c_* are the Poisson’s ratios of the plate and core layer, respectively; *G_c_* is the shear modulus of the core layer; *M_r_*, *M_θ_*, and *M_rθ_* are the bending moment and torque in polar coordinates; *Q_r_* and *Q_θ_* are the transverse shear forces in polar coordinates; *w* is the deflection; and *ψ_r_* and *ψ_θ_* are the turning angles.

The deflection *w* and the turning angle *ψ_r_*, *ψ_θ_* can be expressed by the functions *ω* and *f* as follows:

(5) $$\psi_{r}=\frac{\partial \omega }{\partial r}+\frac{1}{r}\frac{\partial f}{\partial \theta }$$

(6) $$\psi_{\theta }=\frac{1}{r}\frac{\partial \omega }{\partial \theta }-\frac{\partial f}{\partial r}$$

After coordinate transformation, the basic equation for bending of SP-HCs after decoupling in polar coordinates can be obtained as follows:

(7) $$\begin{array}{l} w=\omega -\frac{D+D_{c}}{C}\nabla^{2}\omega \\ \left[\frac{D}{2}\left(1-\mu_{f}\right)+\frac{D_{c}}{2}\left(1-\mu_{c}\right)\right]\nabla^{2}f-Cf=0\\ \left(D+D_{c}\right)\nabla^{2}\nabla^{2}\omega =q \end{array}$$

where $\nabla^{2}$ is the Laplace operator in polar coordinates, $\nabla^{2}=\frac{\partial^{2}}{\partial r^{2}}+\frac{1}{r}\frac{\partial }{\partial r}+\frac{1}{r^{2}}\frac{\partial^{2}}{\partial \theta^{2}}$.

Axisymmetric bending of circular plates is relatively common in practical engineering applications. A circular sandwich plate of radius *a* is subjected to an axisymmetric transverse load *q*, with boundary restraints simply supported or fixed-end supported at the periphery. At this point, the basic equation is as follows:

(8) $$\frac{1}{r}\frac{d}{dr}\left\{r\frac{d}{dr}\left[\frac{1}{r}\frac{d}{dr}\left(r\frac{d\omega }{dr}\right)\right]\right\}=\frac{q}{D+D_{c}}$$

If the transverse load *q* is not a function of the radius *r*, the above equation can be obtained by repeating the integration of *r*:

(9) $$\omega =\frac{qr^{4}}{64\left(D+D_{c}\right)}+\frac{1}{4}Ar^{2}\left(\ln r-1\right)+\frac{1}{4}Br^{2}+E\ln r+F$$

Then, the Laplace operator of ω is

(10) $$\nabla^{2}\omega =\frac{qr^{2}}{4\left(D+D_{c}\right)}+Alnr+B$$

Then, the deflection of the circular plate can be obtained as

(11) $$\begin{array}{cc} w= & \omega -\frac{D+D_{c}}{C}\nabla^{2}\omega =\frac{qr^{4}}{64\left(D+D_{c}\right)}-\frac{qr^{2}}{4C}+\frac{1}{4}Ar^{2}\left(\ln r-1\right)+\\ & \frac{1}{4}Br^{2}+\left(E-\frac{D+D_{c}}{C}A\right)\ln r+F-\frac{D+D_{c}}{C}B \end{array}$$

where the four integration constants, *A*, *B*, *E* and *F*, are unknown quantities. Only when these four integration constants are obtained, can the deflection formula of each point of a circular sandwich plate be determined, so as to determine the internal force and stress magnitude of each point of this circular plate. These four integration constants can be derived from the boundary conditions at the center and edges of the circular plate.

### 2.1. Axisymmetric Bending of Circular and Non-Porous SP-HC

**Figure 2:**
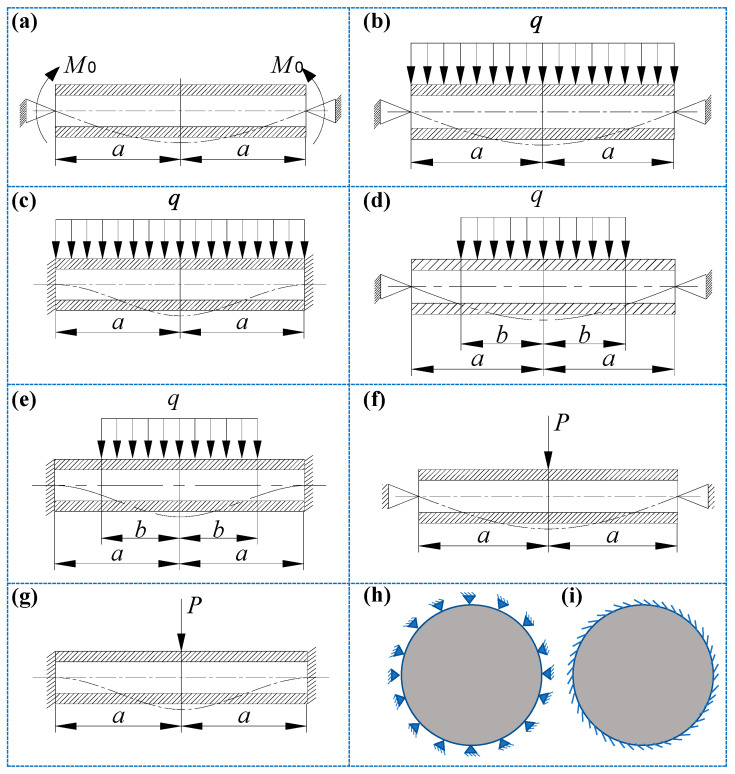
Boundary constraints and loading conditions for circular and non-porous SP-HC. (**a**) Simply supported at the periphery and uniformly distributed bending moment under boundary action; (**b**) simply supported at the periphery, subjected to transverse uniform load; (**c**) fixed-end supported at the periphery, subjected to transverse uniform load; (**d**) simply supported at the periphery, subjected to local transverse uniform load; (**e**) fixed-end supported at the periphery, subject to local transverse uniform load; (**f**) simply supported at the periphery, subject to central concentrated force; (**g**) fixed-end supported at the periphery, subject to central concentrated force; (**h**) the top view of the circular sandwich plate simply supported at its periphery; and (**i**) the top view of the circular sandwich plate fixed-end supported at its periphery.

There is a circular and non-porous SP-HC with radius *a*, plate thickness *t*, and core thickness *h*. The elastic moduli of the plate and core are *E* and *E_c_*, respectively, and the Poisson’s ratios are *μ_f_* and *μ_c_*, respectively. The different boundary constraints and load conditions are given in Figure 2.

#### 2.1.1. The Boundary of the Circular Sandwich Plate Simply Supported at Its Periphery Subjected to the Uniformly Distributed Bending Moment

As shown in Figure 2a, when the periphery of the circular sandwich plate is subjected to the uniformly distributed bending moment *M*_0_ without uniform load in the transverse direction, the deflection formula is

(12) $$w=\frac{M_{0}\left(a^{2}-r^{2}\right)}{2\left[D\left(1+\mu_{f}\right)+D_{c}\left(1+\mu_{c}\right)\right]}$$

#### 2.1.2. Circular Sandwich Plate Subjected to Transverse Uniform Load

(1) Simply supported circular sandwich plate

As shown in Figure 2b, when the circular sandwich plate simply supported at its periphery is subjected to the uniformly distributed load of pressure *q*, the deflection formula is

(13) $$w=\frac{q}{64\left(D+D_{c}\right)}\left(a^{2}-r^{2}\right)\left(\frac{5+\upsilon }{1+\upsilon }a^{2}-r^{2}\right)+\frac{q}{4C}\left(a^{2}-r^{2}\right)$$

where υ is the overall equivalent Poisson’s ratio of the sandwich plate, $\upsilon =\frac{D\mu_{f}+D_{c}\mu_{c}}{D+D_{c}}$.

(2) Fixed-end supported circular sandwich plate

As shown in Figure 2c, when the circular sandwich plate fixed-end supported at its periphery is subjected to the uniformly distributed load of pressure *q*, the deflection formula is

(14) $$w=\frac{q}{64\left(D+D_{c}\right)}{\left(a^{2}-r^{2}\right)}^{2}+\frac{q}{4C}\left(a^{2}-r^{2}\right)$$

#### 2.1.3. Circular Sandwich Plate Subjected to a Uniform Load in a Circle of Radius *b*

(1) Simply supported circular sandwich plate

As shown in Figure 2d, when the sandwich plate simply supported at its periphery is subjected to the uniformly distributed load of strength *q* in a circle of radius *b*, the deflection formula is

(15) $$\begin{array}{ll} w_{1} & =\frac{qr^{4}}{64\left(D+D_{c}\right)}-\frac{qb^{2}r^{2}}{8\left(D+D_{c}\right)}\left[\ln\frac{a}{b}+\frac{1}{1+\upsilon }-\frac{1-\upsilon }{4\left(1+\upsilon \right)}\frac{b^{2}}{a^{2}}\right]\\ & +\frac{qb^{2}}{16\left(D+D_{c}\right)}\left[\frac{3+\upsilon }{1+\upsilon }a^{2}-b^{2}\ln\frac{a}{b}-\frac{7+3\upsilon }{4\left(1+\upsilon \right)}b^{2}\right]-\frac{q}{4C}\left(r^{2}-b^{2}-2b^{2}\ln\frac{a}{b}\right)\;\;r\leq b\\ w_{2} & =\frac{qb^{2}r^{2}}{8\left(D+D_{c}\right)}\left[\ln\frac{r}{a}-\frac{3+\upsilon }{2\left(1+\upsilon \right)}+\frac{1-\upsilon }{4\left(1+\upsilon \right)}\frac{b^{2}}{a^{2}}\right]+\frac{qb^{4}}{16\left(D+D_{c}\right)}\ln\frac{r}{b}\\ & +\frac{qb^{2}}{16\left(D+D_{c}\right)}\left[\frac{3+\upsilon }{1+\upsilon }a^{2}-\frac{1-\upsilon }{2\left(1+\upsilon \right)}b^{2}\right]-\frac{qb^{2}}{2C}\ln\frac{r}{a}\;\;b\leq \;r\leq a \end{array}$$

(2) Fixed-end supported circular sandwich plate

As shown in Figure 2e, when the sandwich plate fixed-end supported at its periphery is subjected to the uniformly distributed load of strength *q* in a circle of radius *b*, the deflection formula is

(16) $$\begin{array}{ll} w_{1} & =\frac{qr^{4}}{64\left(D+D_{c}\right)}-\frac{qb^{2}r^{2}}{32\left(D+D_{c}\right)}\left(4\ln\frac{a}{b}+\frac{b^{2}}{a^{2}}\right)\\ & +\frac{qb^{2}}{16\left(D+D_{c}\right)}\left[a^{2}-b^{2}\left(\frac{3}{4}+\ln\frac{a}{b}\right)\right]+\frac{qb^{2}}{4C}+\frac{qb^{2}}{2C}\ln\frac{a}{b}-\frac{qr^{2}}{4C}r\;\;\leq b\\ w_{2} & =\frac{qb^{2}r^{2}}{8\left(D+D_{c}\right)}\left(\ln\frac{r}{a}-\frac{1}{2}-\frac{b^{2}}{4a^{2}}\right)+\frac{qb^{4}}{16\left(D+D_{c}\right)}\ln\frac{r}{a}\\ & +\frac{qb^{2}}{16\left(D+D_{c}\right)}\left(a^{2}+\frac{b^{2}}{2}\right)-\frac{qb^{2}}{2C}\ln\frac{r}{a}\;\;b\leq r\leq a \end{array}$$

#### 2.1.4. Circular Sandwich Plate Subjected to Central Concentration Force

(1) Simply supported circular sandwich plate

As shown in Figure 2f, when the center of the sandwich plate simply supported at its periphery is subjected to the concentrated load *P*, the deflection formula is

(17) $$w=\frac{P}{16\pi \left(D+D_{c}\right)}\left[\frac{3+\upsilon }{1+\upsilon }\left(a^{2}-r^{2}\right)+2r^{2}\ln\frac{r}{a}\right]-\frac{P}{2\pi C}\ln\frac{r}{a}$$

(2) Fixed-end supported circular sandwich plate

As shown in Figure 2g, when the center of the sandwich plate fixed-end supported at its periphery is subjected to the concentrated load *P*, the deflection formula is

(18) $$w=\frac{P}{16\pi \left(D+D_{c}\right)}\left[a^{2}-r^{2}+2r^{2}\ln\frac{r}{a}\right]-\frac{P}{2\pi C}\ln\frac{r}{a}$$

For the analytical solution of the bending deformation of the circular SP-HC given in this paper, if the bending stiffness of the core layer of the sandwich plate is neglected, i.e., if *D_c_* = 0 is set in the equation, the same deflection expression as that of the circular SP-SC in [2] is obtained. For the circular SP-HC, the bending stiffness *D_c_* of the core layer will make its deflection smaller than that of the circular SP-SC under the same conditions, so the circular SP-HC has a higher load capacity compared with the circular SP-SC.

### 2.2. Axisymmetric Bending of the Annular SP-HC

If the circular sandwich plate has a circular hole at the center of the circle, it becomes an annular plate with two concentric circles at the boundary.

#### 2.2.1. Annular Plates with Uniformly Distributed Bending Moment M_1_ and M_2_ at the Inner and Outer Edges, Respectively

**Figure 3:**
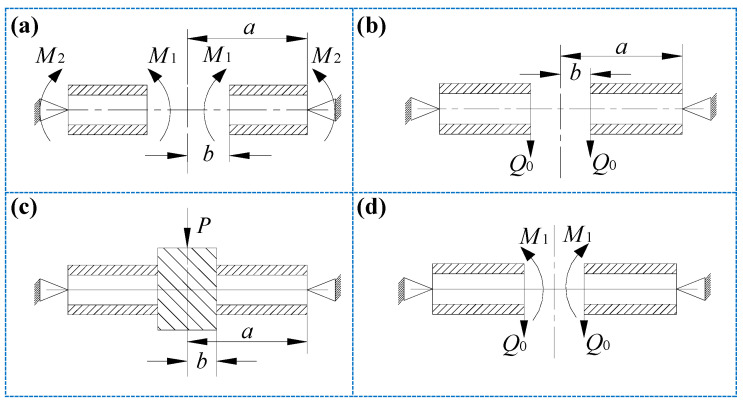
Boundary constraint conditions and loading conditions for annular SP-HC: (**a**) uniformly distributed bending moments *M*_1_ and *M*_2_ acting on the outer boundary simply supported, inner and outer boundaries, respectively; (**b**) simply supported at the outer boundary and uniform shear force *Q*_0_ acting along the inner edge; (**c**) simply supported at the outer boundary and fixed-end supported at the inner edge; (**d**) force diagram of the circular plate simply supported at the outer boundary and fixed-end supported at the inner edge.

As shown in Figure 3a, when uniformly distributed bending moments *M*_1_ and *M*_2_ act on the inner and outer boundaries of the annular plate, and the outer boundary is a simply supported edge, the deflection formula is

(19) $$\begin{array}{ll} w= & \frac{M_{1}b^{2}-M_{2}a^{2}}{2\left(a^{2}-b^{2}\right)\left[D\left(1+\mu_{f}\right)+D_{c}\left(1+\mu_{c}\right)\right]}\left(r^{2}-a^{2}\right)\\ & +\frac{a^{2}b^{2}\left(M_{1}-M_{2}\right)}{\left(a^{2}-b^{2}\right)\left[D\left(1-\mu_{f}\right)+D_{c}\left(1-\mu_{c}\right)\right]}\ln\frac{r}{a} \end{array}$$

#### 2.2.2. Annular Plates with Uniform Shear Force *Q*_0_ Acting along the Inner Edge

As shown in Figure 3b, when the inner edge of the annular plate is subjected to a uniformly distributed shear force *Q*_0_ and the outer boundary is a simply supported edge, the deflection formula is

(20) $$\begin{array}{cc} w= & -\frac{Q_{0}br^{2}}{4\left(D+D_{c}\right)}\left[\ln\frac{r}{b}-\frac{a^{2}}{a^{2}-b^{2}}\ln\frac{a}{b}-\frac{3+\upsilon }{2\left(1+\upsilon \right)}\right]+\frac{Q_{0}b}{2\left(D+D_{c}\right)}\frac{1+\upsilon }{1-\upsilon }\frac{a^{2}b^{2}}{\left(a^{2}-b^{2}\right)}\ln\frac{a}{b}\ln\frac{r}{a}\\ & -\frac{Q_{0}a^{2}b}{4\left(D+D_{c}\right)}\left[\frac{b^{2}}{a^{2}-b^{2}}\ln\frac{a}{b}+\frac{3+\upsilon }{2\left(1+\upsilon \right)}\right]+\frac{Q_{0}b}{C}\ln\frac{r}{a} \end{array}$$

#### 2.2.3. Annular Plates with Fixed-End Support at the Inner Edge

As shown in Figure 3c, the center part of the annular plate *r = b* is regarded as a rigid body, so that the inner edge of the annular plate forms a fixed-end supported boundary condition. The concentrated load *P* acts at the center of the circle, and the outer edge of the annular plate is a simply supported edge. At this time, the force of the annular plate is shown in Figure 3d, and its deflection can be obtained by superposing the expressions of bending moment and shear force shown in Equation (21) into Equations (19) and (20).

(21) $$\begin{array}{l} M_{1}=\frac{P}{4\pi \left[\left(1+\upsilon \right)\frac{a^{2}}{b^{2}}+1-\upsilon \right]}\left[\left(1-\upsilon \right)\left(\frac{a^{2}}{b^{2}}-1\right)+2\left(1+\upsilon \right)\frac{a^{2}}{b^{2}}\ln\frac{a}{b}\right]\\ M_{2}=0\\ Q_{0}=-\frac{P}{2\pi b} \end{array}$$

### 2.3. Analysis of Examples

#### 2.3.1. Bending of Circular SP-HC

There is a circular sandwich plate with a radius of *a* = 150 mm, subjected to a transverse uniform load *q* = 1.0 MPa. The thickness of upper and lower plates *t* = 1 mm, modulus of elasticity *E_f_*= 68 GPa, thickness of cores *h* = 15 mm, and Poisson’s ratio between plates and cores *μ_f_* = *μ*_c_ = 0.3. A comparison of the maximum deflection of the plates under different conditions of elastic modulus of the core layer is shown in Table 1 and Table 2.

According to the above calculation results, when *E_c_*/*E_f_* is less than 1/50, the analytical solutions calculated by the proposed formula in this study, the analytical solution based on Reissner’s theory, and the numerical solution of Nastran software tend to be consistent. Therefore, the core layer can be treated as a soft core when *E_c_*/*E_f_* is less than 1/50, and the bending formula derived in this study for hard cores can be precisely suitable for a circular sandwich plate with soft cores. When *E_c_*/*E_f_* is greater than 1/50, the error increases significantly. The core layer must be treated as a hard core, so it also shows that the analytical solution for the circular SP-HCs derived in this paper is necessary, because the previous bending formula for SP-SC is not suitable for SP-HC, while the present formula proposed in this study can be suitable to SP-HC and SP-SC.

**Figure 4:**
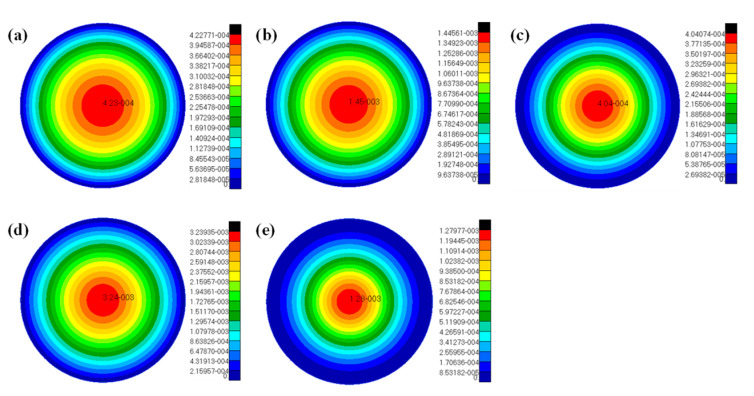
The distribution cloud chart of bending deformation of the circular SP-HC under different loads and boundary constraints: (**a**) simply supported boundary, uniformly distributed bending moment *M*_0_ = 570 N^.^m; (**b**) simply supported boundary, uniform load *q* = 0.5 MPa; (**c**) fixed-end supported boundary, uniform load *q =* 0.5 MPa; (**d**) simply supported boundary, local uniform load *q*_b_ = 4.5 MPa; and (**e**) fixed-end supported boundary, local uniform load *q*_b_ = 4.5 MPa.

For the above calculation example, the elastic modulus *E*_c_ = 6.8 GPa of the core layer is taken, and the analytical and numerical solutions of the maximum deflection of bending for the circular SP-HC under different loads and boundary constraints are calculated, as shown in Table 3. The distribution cloud chart of bending deformation of the circular SP-HC under each condition is shown in Figure 4a–e, respectively.

#### 2.3.2. Bending of Annular SP-HC

Consider an annular SP-HC (circular SP-HC with a hole in the center) with radius *a =* 200 mm; thickness of the upper and lower panels *t =* 1 mm, modulus of elasticity *E_f_*= 68 GPa; thickness of the core layer *h* = 15 mm, modulus of elasticity *E_c_* = 6.8 GPa; and Poisson’s ratio of the panels and core layer *μ_f_* = *μ*_c_ = 0.3. The ratio of the radius a of the annular plate to the radius *b* of the circular hole is denoted by *k*. With the variation of *k* value, the maximum deflections of the simply supported annular sandwich plate with uniformly distributed bending moment *M*_1_ = 400 N^.^m acting at the inner edge and the simply supported annular sandwich plate with uniform shear force *Q*_0_ = 5000 N acting at the inner edge are calculated as shown in Table 4.

From the above analyses, it can be seen that the numerical solution of annular SP-HCs calculated by the finite element software Nastran is very close to the analytical solution derived in this paper, with an error of less than 2%, regardless of the boundary conditions and load forms. This indicates that the analytical solution derived in this paper can be used for the bending analysis of the circular and annular SP-HCs in engineering.

## 3. Analysis of the Overall Stability of Circular SP-HC

The structure should not only ensure sufficient strength and stiffness, but also ensure sufficient stability. The classical linear buckling theory is generally used in the current engineering analysis of structural stability. The most common failure mode of composite sandwich structures when subjected to compression, torsion, shear, and bending loads is instability. Therefore, it is significant to analyze the stability of the sandwich structure. With regard to circular SP-HC, by considering the derivation of the equilibrium equation for the in-plane load action and using a method similar to the bending problem for sandwich plates, the buckling differential equation for SP-HC in polar coordinates can be obtained as follows

(22) $$\left(D+D_{c}\right)\nabla^{2}\nabla^{2}\omega -[N_{\theta }\left(\frac{1}{r}\frac{\partial }{\partial r}+\frac{1}{r^{2}}\frac{\partial^{2}}{\partial \theta^{2}}\right)+N_{r}\frac{\partial^{2}}{\partial r^{2}}+2N_{r\theta }\left(\frac{1}{r}\frac{\partial^{2}}{\partial r\partial \theta }-\frac{1}{r^{2}}\frac{\partial }{\partial \theta }\right)]\left(\omega -\frac{D+D_{c}}{C}\nabla^{2}\omega \right)=0$$

where *N_r_*, *N_θ_*, and *N_rθ_* are the in-plane load in the *r* direction, in-plane load in the *θ* direction, and in-plane shear load in polar coordinates, respectively.

### 3.1. Stability of Circular Sandwich Plate under Uniform Pressure

**Figure 5:**
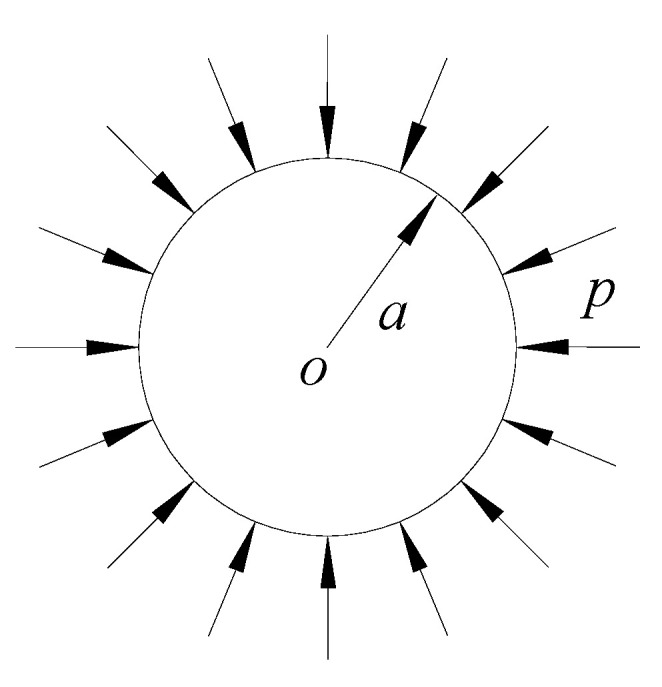
Circular SP-HCs subjected to uniform pressure.

A circular SP-HC with radius *a* under a uniform pressure *p* is shown in Figure 5. Assuming that the boundary is simply supported or fixed-end supported at the periphery, the plate will deform axisymmetrically in the case of instability. According to the analysis of the plane stress problem, the internal forces $N_{r}=N_{\theta }=-p$ and $N_{r\theta }=0$ in the mid-plane can be obtained. By substituting the internal forces into Equation (22), the buckling differential equation of the circular sandwich plate under axisymmetric condition can be obtained as

(23) $$\left(D+D_{c}\right)\nabla^{2}\nabla^{2}\omega +p\left(\frac{1}{r}\frac{d}{dr}+\frac{d^{2}}{dr^{2}}\right)\left(\omega -\frac{D+D_{c}}{C}\nabla^{2}\omega \right)=0$$

Thus, the critical pressure of the circular sandwich plate fixed-end supported at its periphery can be obtained as

(24) $$p_{cr}=\frac{14.68\left(D+D_{c}\right)C}{Ca^{2}+14.68\left(D+D_{c}\right)}$$

The critical pressure of the circular sandwich plate simply supported at its periphery is

(25) $$p_{cr}=\frac{4.2\left(D+D_{c}\right)C}{Ca^{2}+4.2\left(D+D_{c}\right)}$$

### 3.2. Analysis of Example

**Figure 6:**
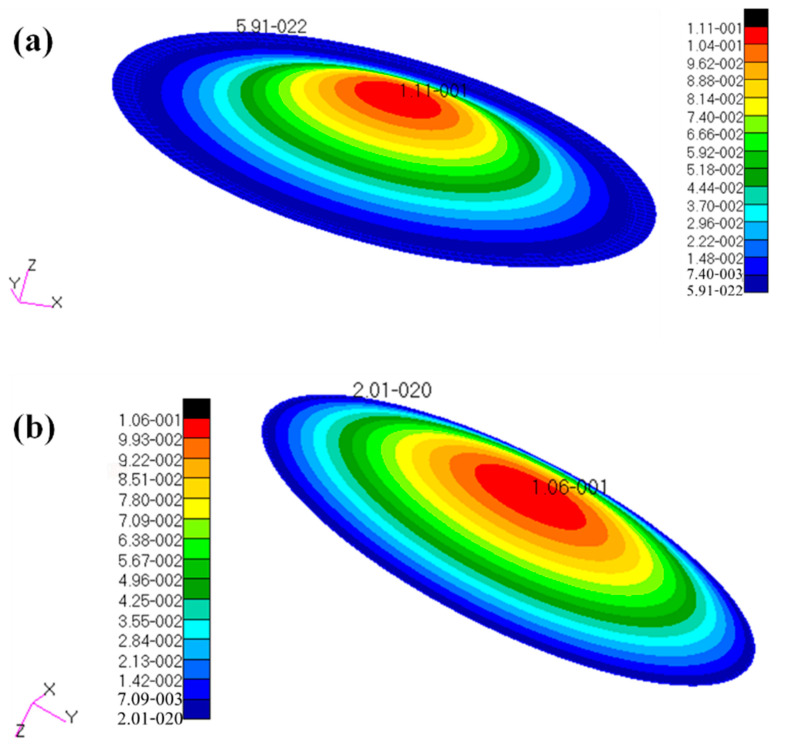
The first-order buckling mode shapes of circular SP-HCs: (**a**) fixed-end supported at the periphery; (**b**) simply supported at the periphery.

There is a circular SP-HC subjected to a uniform pressure *p* in the radius direction with radius *a* = 150 mm, thickness of both upper and lower panels *t* = 1 mm and modulus of elasticity *E_f_* = 68 GPa; thickness of the core layer *h* = 15 mm and modulus of elasticity *E_c_*= 6.8 GPa; and Poisson’s ratio of both plates and core layers *μ_f_* = *μ_c_* = 0.3. The stability analysis was performed on this plate to calculate the critical load for its instability, as shown in Table 5. The numerical calculation results of the finite element software Nastran are given in Table 5 and Figure 6.

By comparative analysis above, the theoretical solutions of the destabilizing critical load of circular SP-HCs under different constraints are in good agreement with the finite element solution, and the relative error does not exceed 1%. This indicates that the theoretical model of stability analysis of circular SP-HCs given in this paper can provide a theoretical basis for the stability analysis of such plate in engineering.

## 4. Conclusions

In this paper, the theoretical and finite element analyses of the bending and stability of circular and annular SP-HCs under different boundary conditions and load forms were conducted for the first time, and the following conclusions can be drawn:

(1) Based on the revisions for Reissner’s sandwich plate theory, the bending equilibrium equations for isotropic circular SP-HC in the polar coordinate system are derived, and the basic set of equations is decoupled and simplified to derive the analytical solutions for the bending deformation of circular and annular sandwich plates under common loads and boundary conditions.
(2) If the bending stiffness of the core layer can be neglected, i.e., if *D_c_*= 0 is set in the equation, the analytical solution of bending deformation of the circular SP-HC derived in this paper can obtain the same deflection expression as that of the circular SP-SC. Theoretical and numerical calculations for some examples show that the theoretical solution proposed in this paper, the theoretical solution of SP-SC, and the finite element solution tend to be in good agreement when the elastic modulus of the core becomes smaller, i.e., the core becomes soft. Therefore, the theory of circular SP-HC derived in this paper can also be used for the calculation of circular SP-SCs, which boasts a wider range of applicability.
(3) The analytical solutions of the bending deformation of the annular SP-HC simply supported at its periphery under two forms of loadings (uniform bending moment and uniform shear force acting along the inner edge) are derived. Then, the variation of the inner edge deflection (maximum deflection) with the value of *k* (the ratio of the outer radius to the inner radius of the annular plate) is discussed in conjunction with the finite element analysis. The results show that the analytical solution and the finite element solution have good agreement. The inner edge deflection increases with the decrease in *k* value (i.e., the size of central hole increases) under the uniformly distributed bending moment load. Under the uniformly distributed shear load, the inner edge deflection increases initially and then decreases with the decrease in *k* value.
(4) The buckling differential equation of the SP-HC in polar coordinates and the solutions of the critical loads for the overall instability of the SP-HC under simply supported and fixed-end supported boundary conditions are derived. Some calculation examples show that the critical loads derived in this paper are in good agreement with the finite element analysis results.

## Figures and Tables

**Table 1:** Maximum deflection of the circular sandwich plate simply supported at its periphery with different modulus of elasticity of the core layer.

E_c_/E_f_	Hc/mm	Re/mm	Na/mm	Hc-Na	Re-Na	Hc-Re
1/1	1.068	3.385	1.069	−0.09%	68.41%	−216.95%
1/2	1.633	3.399	1.634	−0.06%	51.93%	−108.15%
1/5	2.410	3.439	2.407	0.12%	30.01%	−42.70%
1/10	2.899	3.506	2.891	0.28%	17.54%	−20.94%
1/50	3.902	4.044	3.858	1.13%	4.60%	−3.64%
1/100	4.643	4.716	4.556	1.87%	3.39%	−1.57%
1/1000	16.806	16.814	15.932	5.20%	5.24%	−0.04%

Note: “Hc” is the analytical solution of the hard core by the proposed equations in this study; “Re” is the analytical solution based on Reissner’s theory; “Na” is the numerical solution by Nastran software; “Hc-Na” is the error of the analytical solution of the hard core by the proposed equations in this study relative to the numerical solution of Nastran; “Re-Na” is the error of the analytical solution of the hard core based on Reissner’s theory relative to the numerical solution of Nastran; and “Hc-Re” is the error of the analytical solution of the hard core by the proposed equations in this study relative to the analytical solution of Reissner.

**Table 2:** Maximum deflection of the circular sandwich plate fixed-end supported at its periphery with different modulus of elasticity of the core layer.

E_c_/E_f_	Hc/mm	Re/mm	Na/mm	Hc-Na	Re-Na	Hc-Re
1/1	0.272	0.840	0.274	−0.74%	67.38%	−208.8%
1/2	0.421	0.854	0.423	−0.48%	50.47%	−102.9%
1/5	0.642	0.894	0.642	0	28.19%	−39.25%
1/10	0.812	0.961	0.808	0.49%	15.92%	−18.35%
1/50	1.464	1.499	1.425	2.67%	4.94%	−2.39%
1/100	2.153	2.171	2.070	3.86%	4.65%	−0.84%
1/1000	15.163	15.182	14.226	6.18%	6.30%	−0.12%

**Table 3:** Maximum deflection of bending for circular SP-HCs under different loads and boundary constraints.

Boundary Conditions	Load Form and Size	Analytic Solution of *w*_max_/mm	Numerical Solution of *w*_max_/mm	Error
Simply supported boundary	uniformly distributed bending moment *M*_0_ = 570 N·m	0.423	0.423	0
Simply supported boundary	Uniform load *q* = 0.5 MPa	1.449	1.446	0.21%
Fixed-end supported boundary	Uniform load *q* = 0.5 MPa	0.406	0.404	0.49%
Simply supported boundary	Local uniform load *q_b_* = 4.5 MPa	3.263	3.239	0.74%
Fixed-end supported boundary	Local uniform load *q_b_* = 4.5 MPa	1.292	1.280	0.93%

**Table 4:** Maximum deflection of bending of annular SP-HCs simply supported on the outer boundary with a hole in the center under different *k* values.

*k* Value	Analytical Solution of *w*_max_ When the Inner Edge Is Subjected to the Uniformly Distributed Bending Moment *M*_1_ = 400 N·m/mm	Numerical Solution of *w*_max_ When the Inner Edge Is Subjected to the Uniformly Distributed Bending Moment *M*_1_ = 400 N·m/mm	Error/%	Analytical Solution of *w*_max_ When the Inner Edge Is Subjected to the Uniform Shear Force *Q*_0_ = 5000 N/mm	Numerical Solution of *w*_max_ When the Inner Edge Is Subjected to the Uniform Shear Force *Q*_0_ = 5000 N/mm	Error/%
1.25	−1.115	−1.107	0.72	0.543	0.542	0.18
1.5	−0.87	−0.882	1.36	0.689	0.689	0
2	−0.585	−0.577	1.39	0.671	0.67	0.15
3	−0.328	−0.324	1.23	0.491	0.491	0
4	−0.214	−0.218	1.83	0.365	0.364	0.27
5	−0.152	−0.153	0.65	0.285	0.284	0.35

**Table 5:** Critical loads for instability of circular SP-HCs under different constraints.

Boundary Conditions	Analytical Solution	Numerical Solution	Error
Fixed-end supported at the periphery	6.44 × 10^6^ N	6.48 × 10^6^ N	0.62%
Simply supported at the periphery	2.07 × 10^6^ N	2.07 × 10^6^ N	0

## References

[1] Rezaiee-Pajand M., Masoodi A.R., Mokhtari M. (2018). Static analysis of functionally graded non-prismatic sandwich beams. Adv. Comput. Des..

[2] Rezaiee-Pajand M., Mokhtari M. (2020). Size dependent buckling analysis of nano sandwich beams by two schemes. Mech. Adv. Mater. Struct..

[3] Nguyen V.H., Nguyen T.K., Thai H.T., Vo T.P. (2014). A new inverse trigonometric shear deformation theory for isotropic and functionally graded sandwich plates, Compos. Part B Eng..

[4] Vo T.P., Thai H.T., Nguyen T.K., Inam F., Lee J. (2015). Static behaviour of functionally graded sandwich beams using a quasi-3D theory, Compos. Part B Eng..

[5] Phan C.N., Kardomateas G.A., Frostig Y. (2013). Blast response of a sandwich beam/wide plate based on the extended high order sandwich panel theory and comparison with elasticity. J. Appl. Mech..

[6] Rezaiee-Pajand M., Arabi E., Masoodi A.R. (2019). Nonlinear analysis of FG-sandwich plates and shells. Aerosp. Sci. Technol..

[7] Wang X., Yang F., Zeng J., Xiao J. (2007). Design Principles and Applications of Sandwich Structure Composite Materials.

[8] Beijing Institute of Mechanics in Chinese Academy of Sciences (1977). Bending, Stability and Vibration of Sandwich Plate and Shell.

[9] Reissner E. (1948). Finite deflection of sandwich plates. J. Aeronaut. Sci..

[10] Hoff N. (1950). Bendling and buckling of rectangular sandwich panels. Tech. Rep. Arch. Image Libr..

[11] Du Q. (1954). General equations of sandwich plates under transverse loads and edgewise shears and compressions. Acta Physica. Sinica..

[12] Wang Z., Dai F. (1983). Bending, buckling and vibration of orthotropic laminated, sandwiched and stiffened shallow shells. Chin. J. Theor. Appl. Mech..

[13] Thomsen O. (1993). Analysis of local bending effects in sandwich plates with orthotropic face layers subjected to localised loads. Compos. Struct..

[14] Lok T.S., Cheng Q.H. (2001). Bending and forced vibration response of a clamped orthotropic thick plate and sandwich panel. J. Sound Vib..

[15] Lok T.S., Cheng Q.H. (2000). Free vibration of clamped orthotropic sandwich panel. J. Sound Vib..

[16] Pandya B.N., Kant T. (1988). Higher-order shear deformable theories for flexure of sandwich plates—Finite element evaluations. Int. J. Solids Struct..

[17] Sokolinsky V., Frostig Y. (1999). Boundary condition effects in buckling of soft core sandwich panels. J. Eng. Mech..

[18] Heath W.G. (1960). Sandwich Construction-Part II: The Optimum Design of Flat Sandwich Panels. Aircr. Eng..

[19] Aiello M.A., Ombres L. (1997). Local buckling loads of sandwich panels made with laminated faces. Compos. Struct..

[20] Hadi B.K., Matthews F.L. (1998). Predicting the buckling load of anisotropic sandwich panels:an approach including shear deformation of the faces. Compos. Struct..

[21] Frostig Y. (1998). Buckling of sandwich panels with a flexible core-high-order theory. Int. J. Solids Struct..

[22] Dawe D.J., Yuan W.X. (2001). Overall and local buckling of sandwich plates with laminated faceplates, Part I: Analysis. Comput. Methods Appl. Mech. Eng..

[23] Yuan W.X., Dawe D.J. (2001). Overall and local buckling of sandwich plates with laminated faceplates, part II: Applications. Comput. Methods Appl. Mech. Eng..

[24] Pandit M.K., Singh B.N., Sheikh A.H. (2008). Buckling of laminated sandwich plates with soft core based on an improved higher order zigzag theory. Thin-Walled Struct..

[25] Ma C., Deng Z. (2013). Research on bending of simply supported rectangular sandwich plates with hardened cores. Chin. J. Appl. Mech..

[26] Reissner E. (1950). Errata-finite deflection of sandwich plates. J. Aeronaut. Sci..

[27] Deng Z., Ma C., Li D., PI Z. (2014). Study on bending of hard core sandwich plates based on Reissner’s sandwich theory. Sci. Sin. Technol..

